# Prefoldin 5 and Anti-prefoldin 5 Antibodies as Biomarkers for Uveitis in Ankylosing Spondylitis

**DOI:** 10.3389/fimmu.2019.00384

**Published:** 2019-03-05

**Authors:** Oh Chan Kwon, Eun-Ju Lee, Joo Yong Lee, Jeehee Youn, Tae-Hwan Kim, Seokchan Hong, Chang-Keun Lee, Bin Yoo, William H. Robinson, Yong-Gil Kim

**Affiliations:** ^1^Division of Rheumatology, Department of Medicine, Asan Medical Center, College of Medicine, University of Ulsan, Seoul, South Korea; ^2^Asan Institute for Life Science, Asan Medical Center, Seoul, South Korea; ^3^Department of Ophthalmology, Asan Medical Center, College of Medicine, University of Ulsan, Seoul, South Korea; ^4^Department of Anatomy and Cell Biology, College of Medicine, Hanyang University, Seoul, South Korea; ^5^Department of Rheumatology, Hanyang University Hospital for Rheumatic Diseases, Seoul, South Korea; ^6^Division of Immunology and Rheumatology, Stanford University School of Medicine, Stanford, CA, United States

**Keywords:** ankylosing spondylitis, uveitis, prefoldin 5, anti-prefoldin 5 antibody, biomarker

## Abstract

**Objective:** Uveitis is the most common extra-articular manifestation of ankylosing spondylitis (AS), for which no diagnostic biomarkers have been identified. This study was conducted to identify biomarker for uveitis in AS.

**Methods:** To identify autoantibodies associated with uveitis in AS, we performed human protein microarray analysis using sera derived from various autoimmune diseases and ELISA analysis of sera derived from AS and rheumatoid arthritis patients. In the curdlan-induced SKG mice model, ophthalmic examination was performed at week 8 post-immunization and histologic examination of the ocular lesions performed at week 16 post-immunization. Serum levels of target antibodies were assessed at various time-points. To evaluate the functional role of specific autoantibodies, an *in vitro* apoptosis assay using ARPE-19 cells was performed.

**Results:** Reactivity against prefoldin subunit 5 (PFDN5) was identified in AS with uveitis. Levels of anti-PFDN5 antibodies and PFDN5 in sera from AS with uveitis patients were significantly higher than those in AS without uveitis. At week 8, half of curdlan-treated SKG mice developed anterior uveitis, while all of them developed histologically confirmed uveitis at week 16. The levels of anti-PFDN5 antibodies increased over time in the sera of curdlan-treated SKG mice along with increased expression of PFDN5 and apoptosis in the ocular lesions. Knockdown of *PFDN5* in ARPE19 cells resulted in increased apoptosis, suggesting a protective role of PFDN5 against cell death in uveitis.

**Conclusion:** AS patients with uveitis have increased levels of anti-PFDN5 antibodies, and our findings suggest that anti-PFDN5 antibodies could provide a biomarker for uveitis in AS.

## Introduction

Ankylosing spondylitis (AS) is an inflammatory disorder, characterized by both articular and extra-articular manifestations (1). Non-infectious sterile uveitis is the most common extra-articular manifestation of AS (lifetime prevalence: 30–40%) (2). Uveitis in AS patients typically presents as abrupt conjunctival injection, pain, photophobia and visual impairment (3). However, it is difficult to suspect uveitis in patients who have mild or atypical ocular symptoms (4). Moreover, a definitive diagnosis of uveitis necessitates ophthalmologic examination, which may not be readily available. Although, HLA-B27 positivity is a known risk factor for uveitis (5), it is unsuitable for use as a biomarker. Currently there are no reliable biomarkers for uveitis in patients with AS. Identification of such a biomarker may be helpful.

SKG mice are suitable for establishing an animal model of chronic autoimmune arthritis (6). These mice possess a mutation in ZAP-70 (a key signal transducing molecule in T-cells), which results in thymic-positive selection and failure of negative selection of highly self-reactive T-cells including potentially arthritogenic T-cells (6, 7). These self-reactive T-cells lead to chronic arthritis and extra-articular manifestations (6, 8). Curdlan-treated SKG mice develop characteristics of spondyloarthritis including vertebral inflammation, sacroiliitis, peripheral arthritis, and uveitis (9). Therefore, curdlan-treated SKG mice is a good animal model for investigation of uveitis in AS.

Here, we performed human protein microarray analysis of sera from patients with AS or other autoimmune diseases to identify biomarkers for uveitis in AS. We also conducted an animal study using curdlan-treated SKG mice to further evaluate the pathogenic role of the identified biomarker of uveitis in AS.

## Materials and Methods

### Human Samples

ProtoArrays V4 (Invitrogen) were used to profile the autoantibodies present in Multiple Autoimmune Disease Genetics Consortium (MADGC) cohort sera. Arrays were scanned using a GenePix4000B Scanner and median pixel intensities were determined using GenePix Pro software version 5.0 (Axon Instruments). Sera from AS cohort at Hanyang university hospital (Seoul, South Korea) were collected and sera from age- and sex-matched healthy controls and rheumatoid arthritis (RA) patients were collected at the Asan Medical Center (Seoul, South Korea). This study was approved by the Institutional Review Board of Asan Medical Center, Seoul, South Korea (IRB No: 2015-0274) and Hanyang University hospital, Seoul, South Korea (IRB No: 2016-08-006).

### Animal Study

SKG mice with the BALB/c background were obtained from Dr. S. Sakaguchi (University of Kyoto, Japan) (6). Mice were maintained in specific pathogen-free conditions. All mice were handled in accordance with the guidelines for animal care approved by the Institutional Animal Care and Use Committee of Asan Institute for Life Sciences (2015-14-135). Female mice (8 week-old) were administered intraperitoneal injection of 3 mg curdlan suspended in 0.2 mL PBS or 0.2 mL PBS alone (control). Ophthalmic examination was performed at 8 weeks after curdlan injection using surgical microscope. Blood samples were obtained by eye bleeding and sera were separated by centrifugation.

### Enzyme-Linked Immunosorbent Assay (ELISA)

Serum prefoldin subunit 5 (PFDN5) was measured with commercially available ELISA kit (Product No. SEE738Hu, USCN Life Science Inc., USA). Anti-PFDN5 was also measured by ELISA. Ninety-six well plates were coated with 1 μg/mL of recombinant human PFDN5 (Creative Biomart). Diluted serum (1:200) were added and were incubated for 2 h, followed by addition of secondary antibodies (goat anti-human IgG, Jackson ImmunoResearch). Optical density was measured at 450 nm absorbance.

### PET-MRI Scan

At 16 weeks post-injection, whole-body sequential PET/MRI scanning was performed using a nanoScan PET/MRI (Mediso Ltd.). 18F-FDG (0.2 mCi/kg) was injected into the tail vein after a fasting period of at least 12 h, and a 30-min scan was initiated at 40 min after injection of the radioligand. After the MRI scan for 20 min, PET scan was performed for 10 min. MRI scans were acquired, and contiguous axial slices (1 mm) were obtained for the whole body. Scanning parameters were repetition time = 25 s, effective echo time = 3.4 ms, field of view = 64 mm, number of excitations = 1, frequency = 128, and phase = 128. Dynamic data acquisition of PET scans was performed 60–70 min after 18F-FDG injection. Acquired PET images were reconstructed using 3D full detector mode with MRI-based attenuation collection, with an energy level of 250–750 keV and 0.5-mm voxel size.

### Histology

Sixteen weeks after curdlan injection (experimental end-point), eyeball tissues from control and treated mice were fixed in 10% buffered formalin and paraffin-embedded. Sections (4 μm in thickness) were cut and stained with hematoxylin and eosin. For detection of apoptosis and necrotic cell death in eyeball tissues, *in situ* BrdU-Red DNA Fragmentation assay kit (abcam) was used according to the manufacturer's instructions. Stained slides were scanned with the multispectral Vectra scanner (Perkin Elmer).

### FACS Analysis: Apoptosis Assay

ARPE-19, retinal pigment epithelial cells were maintained in DMEM/F12 (Thermo) with 10% FBS and 1% penicillin streptomycin. PFDN5 was silenced using small interfering RNA (siRNA). Cells were transfected with PFDN5 siRNA or scrambled RNA (Thermo) for 24 h using the RNA MAXi transfection reagent (Thermo) according to the manufacturer's instructions. Considering that endoplasmic reticulum (ER) stress is implicated in the pathogenesis of AS (3), we used tunicamycin as an apoptotic stimulus, to induce ER stress induced apoptosis (10). After treatment with Tunicamycin, apoptotic cells were detected with FITC Annexin V Apoptosis Detection kit with 7-AAD according to the manufacturer's instructions.

### Statistical Analysis

All analyses were performed using GraphPad Prism 5 software (GraphPad Software). Receiver operating characteristic curve analysis was performed to evaluate cut-off value of the particular biomarker and its area under the curve. Comparison among different groups were analyzed using Kruskal–Wallis test and ANOVA for non-normal distribution and normal distribution, respectively. Between-group differences (*post-hoc* analysis) were assessed using Mann–Whitney *U*-test and *t*-test for non-normal distribution and non-normal distribution, respectively. Normality was assessed by Kolmogorov–Smirnov test. *P* < 0.05 were considered statistically significant.

## Results

### Identification of an Autoantibody Biomarker for AS With Uveitis

**Figure 1:**
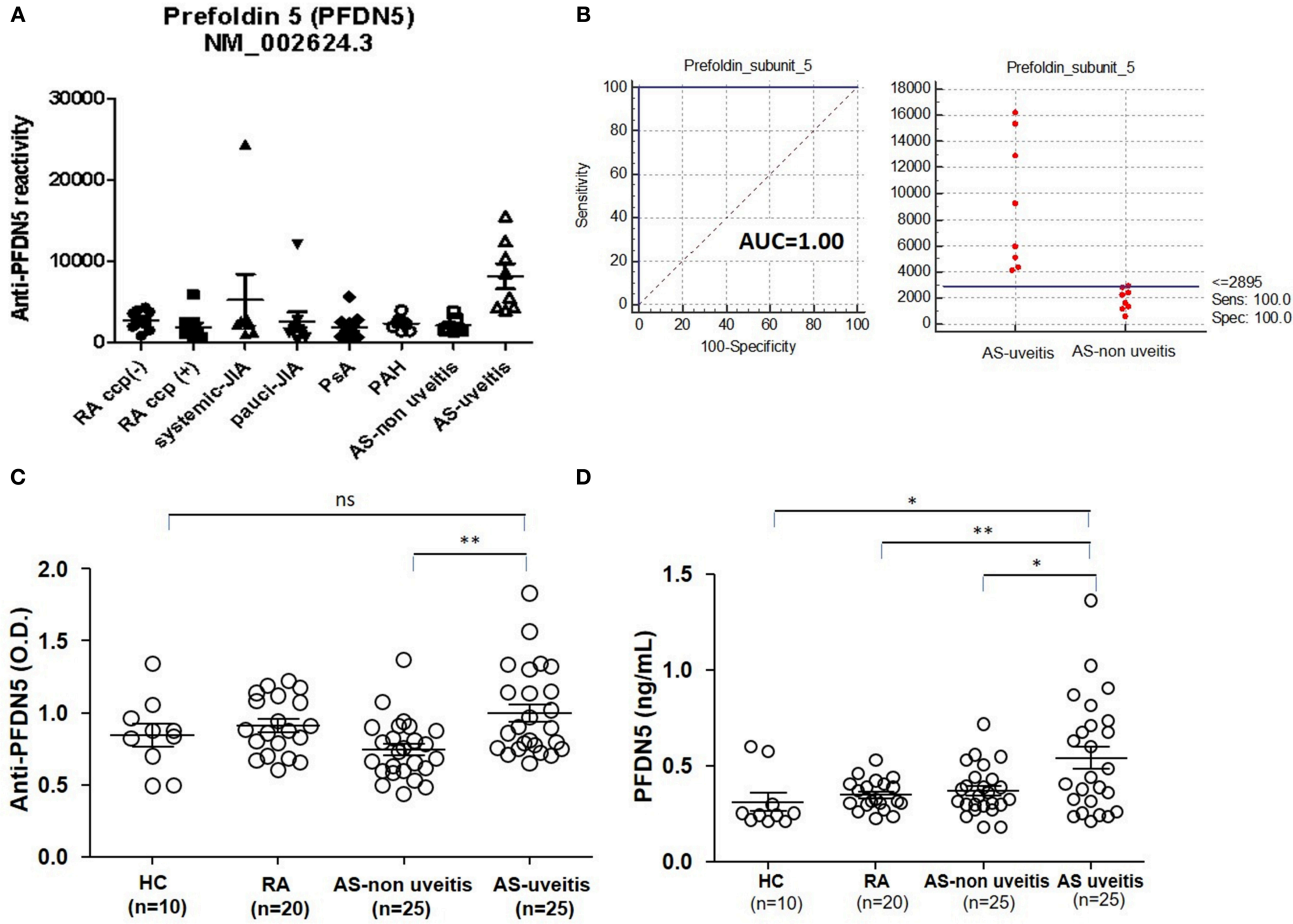
Association between anti-PFDN5 antibody and AS with uveitis. **(A)** Protein microarray analysis of autoreactivity to PFDN5 in sera from patients with various autoimmune diseases in the Multiple Autoimmune Disease Genetics Consortium Cohort. **(B)** Receiver operating characteristic curve for anti-PFDN5 as a biomarker of uveitis in AS. **(C)** ELISA analysis of anti-PFDN5 antibody levels in sera from Korean cohort. **(D)** ELISA analysis of PFDN5 protein levels in sera from Korean cohort. Data presented as mean ± SEM. ^*^p < 0.05; ^**^p < 0.01, by Mann–Whitney *U*-test. PFDN5, prefoldin subunit 5; RA, rheumatoid arthritis; CCP, cyclic citrullinated protein; JIA, juvenile idiopathic arthritis; PsA, psoriatic arthritis; PAH, pulmonary artery hypertension; AUC, area under the curve; HC, healthy control; ELISA, enzyme-linked immunosorbent assay; ns, not significant.

To investigate autoantibody reactivity in sera of patients with various autoimmune diseases in the US-based MADGC cohort consisting of RA (*n* = 21), juvenile idiopathic arthritis (*n* = 15), psoriatic arthritis (*n* = 34), pulmonary arterial hypertension (*n* = 34), AS without uveitis (*n* = 8), AS with uveitis (*n* = 8), we used high-density protein microarrays containing 8,087 human proteins. Increased antibodies to PFDN5 was detected in sera derived from AS patients with uveitis as compared to sera from patients with other diseases, including AS patients without uveitis (Figure 1A). Interestingly, the area under curve of anti-PFDN5 reactivity (cut-off value: 28.95) was 1.00, when compared according to the presence of uveitis in AS patients (Figure 1B). To confirm this finding, we assessed the levels of anti-PFDN5 levels in a cohort of Korean patients consisting of AS with uveitis (*n* = 25), AS without uveitis (*n* = 25), RA (*n* = 20), and healthy control (*n* = 10). Levels of anti-PFDN5 antibodies in AS patients with uveitis were higher than those in AS patients without uveitis (Figure 1C). Considering that PFDN5 is an intracellular protein, we assessed the serum concentration of PFDN5 to evaluate whether it might be released into the extracellular compartment in AS with uveitis. Serum concentrations of PFDN5 protein in AS patients with uveitis were higher than those in other patients (including AS without uveitis) (Figure 1D).

### Elevated Expression of PFDN5 in Retina of Curdlan-Treated SKG Mice With Uveitis

**Figure 2:**
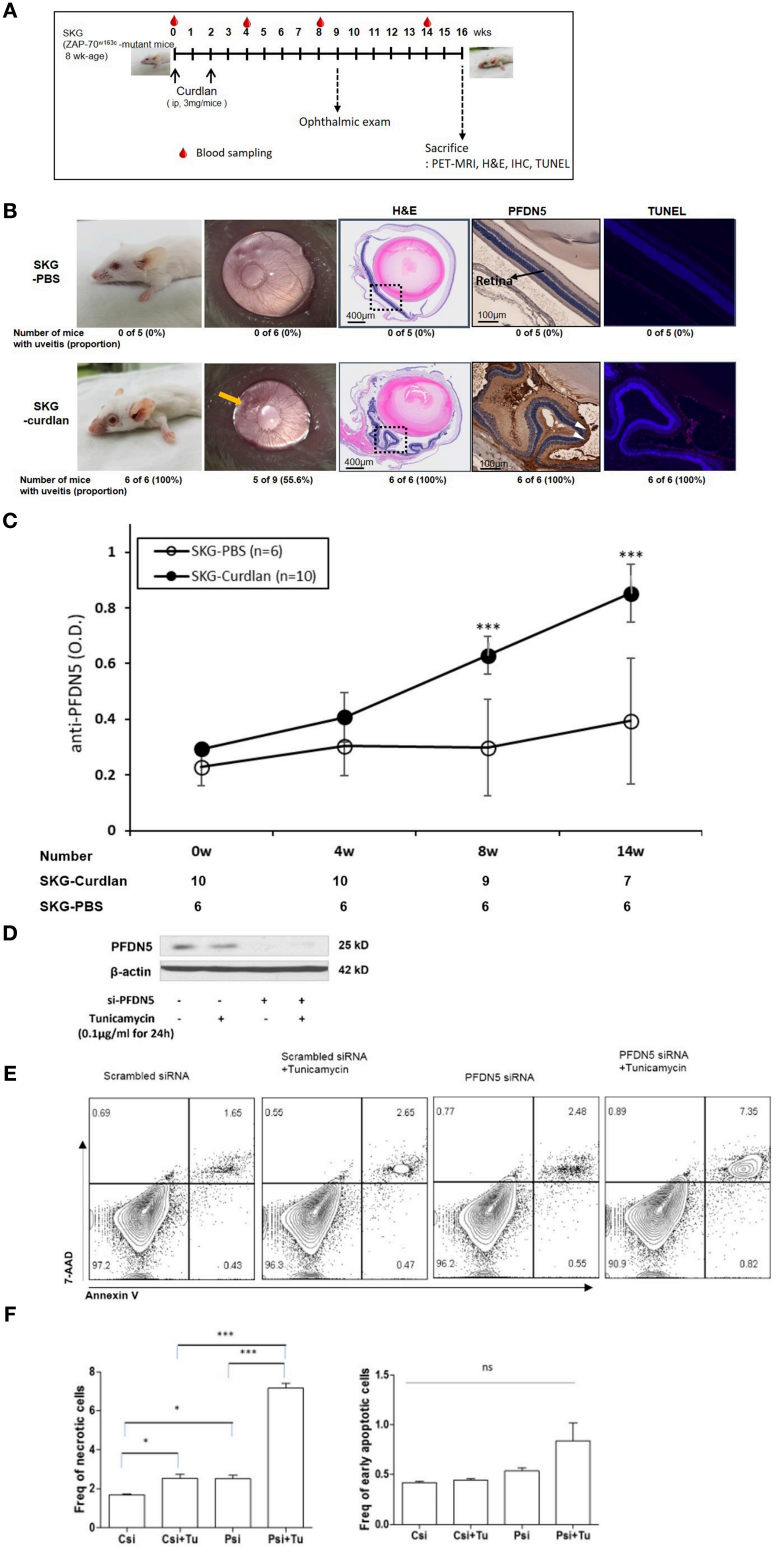
Expression of PFDN5 in curdlan-treated SKG mice with ocular lesions, and the role of PFDN5 against apoptosis in ARPE19 cells. **(A)** The experimental design. **(B)** Comparison of gross phenotype, microscopic ophthalmologic exam, H&E staining, IHC, and TUNEL assay between PBS-treated SKG mice and curdlan-treated-SKG mice. **(C)** ELISA analysis of anti-PFDN5 antibody levels in sera of PBS-treated SKG mice and curdlan-treated SKG mice according to the number of weeks post-injection. **(D)** Western blot showing protein expression of PFDN5 in ARPE19 cells transfected with non-targeting scrambled siRNA, scrambled siRNA with tunicamycin stimulation, siRNA against *PFDN5*, and si-*PFDN5* with tunicamycin stimulation. **(E)** Representative flow cytometry plot of 7-AAD and annexin V staining for ARPE19 cells transfected with non-targeting scrambled siRNA, scrambled siRNA with tunicamycin stimulation, siRNA against *PFDN5*, and si-*PFDN5* with tunicamycin stimulation. **(F)** Comparison of the proportion of necrotic cells or early apoptotic cells among ARPE19 cells transfected with scrambled siRNA, scrambled siRNA under tunicamycin stimulation, *si-PFDN5* and *si-PFDN5* under tunicamycin stimulation. Data presented as mean ± SEM. ^*^p < 0.05; ^***^p < 0.001, by Mann–Whitney *U*-test. H&E, hematoxylin and eosin; PFDN5, prefoldin subunit 5; IHC, immunohistochemistry; TUNEL, Terminal deoxynucleotidyl transferase dUTP Nick-End Labeling; ELISA, enzyme-linked immunosorbent assay; ARPE19, retinal pigment epithelial 19; 7-AAD, 7-aminoactinomycin D; ns, not significant.

We next examined curdlan-treated SKG mice, to determine whether PFDN5 is directly associated with the development of uveitis in AS. The experimental design is shown in Figure 2A. Compared with PBS-treated SKG mice, curdlan-treated SKG mice developed both peripheral arthritis and uveitis (Figure 2B). At week 8 post-injection, microscopic ophthalmologic exam revealed dilated and engorged blood vessels in iris (Figure 2B, yellow arrow) of 5 of 9 curdlan-treated SKG mice, which was compatible with anterior uveitis; however, these findings were not observed in PBS-treated SKG mice (*n* = 6). Histologic exam was performed in 6 curdlan-treated SKG mice and 5 PBS-treated SKG mice at week 16 post-injection, because 4 curdlan-treated and 1 PBS-treated SKG mouse died before sacrifice. On H&E staining of orbital tissues, 6 of the 6 curdlan-treated SKG mice (100%) showed inflammation in ciliary body and in retina. In addition, damage in outer segment of photoreceptors extending to the inner layer of the retina was observed (Figure 2B, box). In contrast, none of the 5 PBS-treated SKG mice (0%) showed evidence of uveitis. Immunohistochemical staining revealed elevated expression of PFDN5 in the retina of curdlan-treated SKG mice (Figure 2B, white arrowhead). Further, increased apoptosis of retinal cells was detected in inner layer of the retina using TUNEL assay, which localized similarly with PFDN5 stained lesions. Level of anti-PFDN5 antibodies in the curdlan-treated SKG mice was significantly higher than that in the PBS-treated SKG mice (Figure 2C). This difference was observed to be statistically significant starting 8 weeks post-injection, which was the time-point at which uveitis became evident in half of the curdlan-treated SKG mice. Representative PET-MRI images are presented in Supplementary Figure 1. In the curdlan-treated SKG mice, hypermetabolic lesions were observed in bilateral orbital area (Supplementary Figure 1, white arrow), which were not detected in PBS-treated SKG mice.

### PFDN5 Protects Retinal Cells From Apoptosis in Uveitis

Based on increased PFDN5 in retinal cells of curdlan-treated SKG mice, we next investigated the functional role of PFDN5 in uveitis. As apoptosis was also observed to be increased in the retinal cells, we focused on the role of PFDN5 in apoptosis. We evaluated the proportion of apoptotic cells among ARPE19 cells under different conditions (scrambled siRNA, scrambled siRNA + tunicamycin, *PFDN5* siRNA, and *PFDN5* siRNA + tunicamycin). Western blot revealed decreased expression of PFDN5 in ARPE19 cells transfected with *PFDN5* siRNA (Figure 2D). A representative flow cytometry plot is shown in Figure 2E. The frequency of necrotic cells (7-AAD^+^Annexin V^+^ cells) in *PFDN5* knockdown cells was significantly higher than that in control. Moreover, under tunicamycin stimulation, we observed a higher frequency of necrotic cells (7-AAD^+^Annexin V^+^ cells) among *PFDN5* knockdown as compared to control cells (Figure 2F). These findings suggest a protective effect of PFDN5 in retinal cells.

## Discussion

In this study, we found elevated serum levels of anti-PFDN5 antibodies and PFDN5 in AS patients with uveitis, whereas the levels were low in AS patients without uveitis. Further, curdlan-treated SKG mice exhibited increased expression of PFDN5 in the ocular lesions and higher serum levels of anti-PFDN5 autoantibody as compared to those in PBS-treated SKG mice; these findings support a potential utility of anti-PFDN5 antibody as a biomarker for uveitis in AS. At week 8 post-injection, uveitis was not evident in some of the curdlan-treated SKG mice, while at week 16 post-injection, uveitis was present in all curdlan-treated SKG mice. Thus, higher level of anti-PFDN5 antibody can be used as a predictor of future development of uveitis. This study is the first to identify a candidate biomarker for the future development of uveitis in AS patients.

To assess the possibility of PFDN5 and anti-PFDN5 as a biomarker of disease activity in AS, we also analyzed the correlation of PFDN5 and anti-PFDN5 antibody with Bath Ankylosing Spondylitis Disease Activity Index (BASDAI) in the Korean cohort (data not shown in the Results). Both PFDN5 (*r* = 0.035, *p* = 0.843) and anti-PFDN5 antibody (*r* = 0.127, *p* = 0.473) did not show any correlation with BASDAI. Thus, we suggest PFDN5 and anti-PFDN5 antibody as biomarkers of uveitis in AS rather than biomarkers of disease activity in AS.

We also observed a protective role of PFDN5 against apoptosis of retinal cells. PFDN is a hexameric protein that is exclusively found in archaea and eukaryotes (11, 12). It is expressed in a wide variety of tissues, including neuronal cells (13). Importantly, genetic disruption of *PFDN5* in mice causes retinal degeneration (13). Functionally, PFDNs bind to and stabilize the unfolded target polypeptides and subsequently deliver them to group II chaperonins (molecular chaperone) to complete the folding process and prevent misfolding of the newly synthesized polypeptides (14). In addition, PFDN also protects the cells from aggregated protein-induced cell death (15). Our findings are consistent with the previous report in which PFDN5 was shown to protect against cell death (15). Based on results of the present study and the known protective function of PFDN5 against cell death, we speculate that expression of PFDN5 is increased in retinal cells, in response to apoptosis resulting from uveitis, to protect from further apoptosis.

In our curdlan-treated SKG mice, histologic examination revealed inflammation as a pan-uveitis including both of anterior chamber and posterior segment. Although panuveitis may occur in some AS patients (16), typical uveitis in AS patients usually involves the anterior chamber (3, 5). Uveitis in curdlan-treated SKG mice is also known to develop in the anterior chamber rather than in the posterior chamber (9). Indeed, at week 8 post-injection, anterior uveitis was observed in curdlan-treated SKG mice. Involvement of anterior chambers and retina at week 16 was likely attributable to extension of inflammation from the anterior chamber to the posterior segment. Therefore, histologically identified uveitis in our model can be considered as an extra-articular manifestation.

Our study has several limitations. First, we lack data regarding the severity of uveitis in the cohort patients at the time of sampling. These data, if were present might have provided the correlation between levels of anti-PFDN5 antibodies and severity of uveitis. Second, in the animal model, 5 curdlan-treated SKG mice had mild uveitis whereas none of the curdlan-treated SKG mice had moderate-to-severe uveitis on ophthalmologic exam at 8 weeks post-injection. Therefore, we were also unable to evaluate the correlation between levels of anti-PFDN5 antibodies and severity of uveitis in the animal model. Third, as non-AS uveitis patients were not included in both the US and Korean cohort, it is not clear whether anti-PFDN5 antibody is also specific to non-AS uveitis. However, our purpose was to identify biomarkers for uveitis, particularly in AS patients. For this purpose, we considered the difference in levels of anti-PFDN5 antibodies between AS patients with uveitis and AS patients without uveitis meaningful.

In conclusion, we demonstrated increased levels of anti-PFDN5 autoantibodies in AS patients with uveitis. We also showed increased expression of PFDN5 in ocular lesions of curdlan-treated SKG mice, and demonstrate a protective role of PFDN5. Our data suggest anti-PFDN5 antibodies as a potential biomarker for diagnosis or prediction of uveitis in AS.

## Author Contributions

WR and Y-GK designed the study. E-JL, WR, and Y-GK performed the experiments. OK, E-JL, JL, T-HK, SH, C-KL, BY, WR, and Y-GK analyzed the data. JY provided the SKG mice. OK, E-JL, WR, and Y-GK wrote the manuscript.

### Conflict of Interest Statement

The authors declare that the research was conducted in the absence of any commercial or financial relationships that could be construed as a potential conflict of interest.

## Supplementary Material

Supplementary Figure 1 PET-MRI performed at week 16 post-injection revealing inflammation in both eyes (more severe in the left side).
