# Supplementary figures and images for: Prefoldin 5 and Anti-prefoldin 5 Antibodies as Biomarkers for Uveitis in Ankylosing Spondylitis
Source: Front Immunol. 2019 Mar 5;10:384. doi: 10.3389/fimmu.2019.00384 (PMC6411661; doi:10.3389/fimmu.2019.00384)

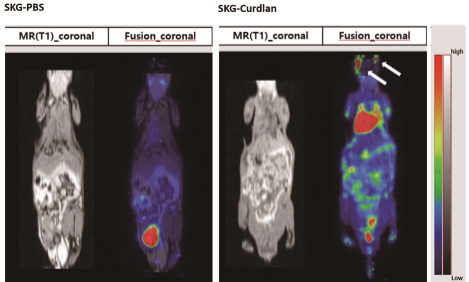

Supplement: Supplementary Figure 1 — PET-MRI performed at week 16 post-injection revealing inflammation in both eyes (more severe in the left side). [file Image_1.TIF]
